# Tissue-resident macrophages co-develop with myocardial tissue in human induced pluripotent stem cell-derived organoids

**DOI:** 10.3389/fcell.2025.1629988

**Published:** 2025-11-13

**Authors:** Anna Frederike Rockel, Tobias Brunnbauer, Nicole Wagner, Brenda Gerull, Süleyman Ergün, Philipp Wörsdörfer

**Affiliations:** 1 Institute of Anatomy and Cell Biology, University of Würzburg, Würzburg, Germany; 2 Comprehensive Heart Failure Center (CHFC) and Department of Internal Medicine I, University Hospital Würzburg, Würzburg, Germany; 3 Atlas University Research Center, Atlas University Istanbul, Istanbul, Türkiye

**Keywords:** tissue resident macrophages, erythrocytes, hematopoiesis, induced pluripotent stem cells (iPSCs), cardiac organoid, tissue model, vascularisation, hemogenic endothelium

## Abstract

The heart is the first functional organ to develop during embryogenesis, forming in parallel with the vasculature and hematopoietic cell lineages. To advance our understanding of human cardiac development and disease, human induced pluripotent stem cell (iPSC)-derived cardiomyocytes offer a promising *in vitro* model. However, conventional 2D cultures lack the complexity required to recapitulate the intricate interactions of different cell types leading to fully functional and mature cardiac tissue. Here, we present a human iPSC-derived 3D organoid model that develops a functional myocardium composed of cardiomyocytes and fibroblasts, capable of spontaneous rhythmic contractions. The organoid is interspersed with a branched network of endothelium-lined cavities, endothelial cords and capillary-like structures. Additionally, hemogenic endothelium co-develops with the cardiac tissue, giving rise to erythrocytes and CCR2^-^ tissue-resident macrophages that integrate into the myocardium. The model represents a complex 3D cell culture platform to study human heart tissue development with all the involved cell types (cardiomyocytes, fibroblasts, endothelial cells, macrophages), paving the way for new insights into the role of macrophages in cardiac development and disease.

## Introduction

In recent years, several human induced pluripotent stem cell (iPSC)-derived self-organizing cardiac organoid models were developed induced by biphasic modulation of the WNT-signaling pathway (Hofbauer et al., 2021; Lewis-Israeli et al., 2021; Drakhlis et al., 2021; Volmert et al., 2023). These models generate different cell types of the heart, such as cardiomyocytes, fibroblasts, endothelial cells, epicardial cells, and additional non-cardiac tissues like foregut epithelium. However, the presence of tissue-resident macrophages has not been described yet.

Macrophages are a crucial component of the heart *in vivo*. They play a role in cardiac development (Xu et al., 2024), maintain cardiac tissue homeostasis (Nicolas-Avila et al., 2020), support electric conduction (Hulsmans et al., 2017), and contribute to the repair and regeneration of cardiac tissue (Zaman and Epelman, 2022). Moreover, recent studies have demonstrated improved contractile force, tissue functionality, and long-term vascularization when macrophages are incorporated into engineered heart tissues (Hamidzada et al., 2024; Landau et al., 2024; Lock et al., 2024).

Therefore, macrophages, included into cardiac organoids, would create a more physiologic model that better mimics the complex interactions within the heart.

In the embryo, cardiac tissue resident macrophages mostly derive through endothelial-to-hematopoietic transition (EHT) which occurs e.g., in the yolk sac and the aorta-gonad-mesonephros region and colonize the fetal heart. The endocardial endothelium in the heart tube has been described as an additional site with hemogenic endothelium (Nakano et al., 2013) which may contribute to a subset of cardiac macrophages (Shigeta et al., 2019). However, this finding is controversially discussed (Liu et al., 2022). Organoids with innately developing macrophages could serve as a platform to investigate the origin of human tissue resident macrophages as well as their role in cardiogenesis.

In addition to enabling the generation of realistic and mature cardiac tissue models or elucidating the developmental origin of cardiac macrophages, cardiac organoids that incorporate macrophages and other immune cells could serve as valuable tools for investigating pathological conditions such as inflammatory cardiomyopathy (Musigk et al., 2024) and immunotherapy-associated cardiotoxicity (Kong et al., 2024).

Here, we describe the co-development of macrophages and myocardial tissue in an organoid model, an aspect that, to our knowledge, has not been reported before. We present evidence that macrophages develop from hemogenic endothelium within endothelium-lined cavities and integrate into the cardiac muscle. They display a tissue-resident macrophage-like gene expression profile as determined by single-cell RNA sequencing. In addition to macrophages, erythrocytes also develop and accumulate within the endothelium-lined cavities of the organoid.

The described organoids offer an advanced platform for studying human cardiogenesis, vascular development, and the involvement of macrophages, in these processes. Unlike previous models that often rely on exogenous addition of macrophages or lack them entirely, this protocol supports their innate emergence. This adds a new layer of physiological relevance and complexity.

## Materials and methods

### Human iPSC culture

All experiments were conducted using the human induced pluripotent stem cell (iPSC) line NHDF iPSC. The reproducibility of cardiac organoid generation and innate macrophage development was confirmed using an independent iPSC line, KK iPSC (Supplementary Figure S2). NHDF iPSCs were generated from commercially available male juvenile human dermal fibroblasts (juvenile NHDF, C-12300, PromoCell) via reprogramming with the hSTEMCCA lentiviral construct (Sommer et al., 2012). KK iPSCs were derived from dermal fibroblasts isolated from a postmortem skin biopsy of a 94-year-old female donor who had consented to body donation through our anatomical institute. These fibroblasts were reprogrammed using a commercially available Sendai virus-based reprogramming kit (CytoTune 2.0 Sendai Reprogramming Vectors, Ref# A16517; Lot# A16517, Invitrogen).

Human iPSCs were cultured on Matrigel-coated 6-well plates in StemMACS iPS-Brew medium (Miltenyi Biotec). Cells were passaged upon reaching approximately 80% confluency using Accutase. Following passaging, cells were reseeded at the desired density in StemMACS iPS-Brew medium supplemented with 10 µM ROCK inhibitor Y-27632.

### Generation of cardiac organoids

To generate cardiac organoids, a single-cell suspension of iPSCs was prepared using Accutase, and 10,000 cells were seeded per well into a round-bottom, ultra-low attachment 96-well plate in 100 µL of StemMACS iPS-Brew medium (Miltenyi Biotec), supplemented with 10 µM ROCK inhibitor Y-27632 (designated as day −1). The plate was incubated in a humidified incubator at 37 °C with 5% CO_2_ on an orbital shaker for 24 h. By day 0, single iPSC aggregates had formed in each well.

To initiate differentiation, 20 µL of medium was removed from each well and replaced with 166.6 µL of mesoderm induction medium (MIM), consisting of RPMI 1640 (Gibco, Cat# 11875-093) supplemented with 2% B27 without insulin (Life Technologies), 6 µM CHIR99021 (Sigma-Aldrich), 1.875 ng/mL BMP4 (PreproTech), and 1.5 ng/mL Activin A (Miltenyi Biotec).

In the following steps, 2/3 (166.5 µL) of the medium were removed per medium change and replaced by new medium.

After 24 h (day 1), the medium was changed to Basic Medium 1 (BM1), composed of RPMI 1640 with 2% B27 without insulin, and incubated for another 24 h. On day 2, to promote cardiac mesoderm specification, the medium was replaced with Cardiac Specification Medium (CSM), consisting of RPMI 1640 supplemented with 2% B27 without insulin and 3 µM IWP2 (Miltenyi Biotec). Aggregates were maintained in CSM for 48 h (until day 4), followed by a medium change back to BM1 for an additional 48 h.

From day 6 onward, for cardiac maturation and maintenance, the medium was switched to Basic Medium 2 (BM2), composed of RPMI 1640 supplemented with 2% B27 (Life Technologies). BM2 was refreshed every other day until the organoids were either fixed for histological sectioning, dissociated for single-cell RNA sequencing, or subjected to calcium imaging or other analyses.

**FIGURE 1:**
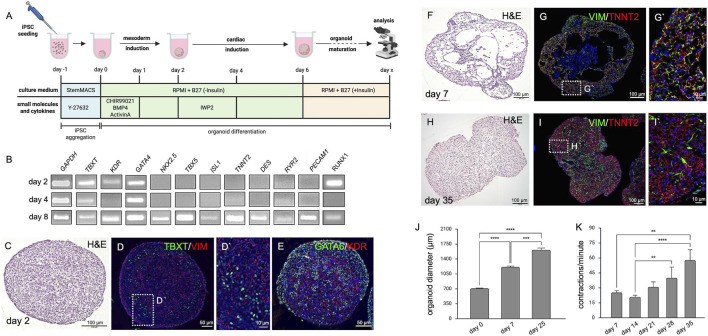
Cardiac Organoids. **(A)** Schematic representation of the cardiac organoid differentiation protocol displaying experimental timing, cell culture media as well as small molecules and cytokines used to induce cardiac tissue. The image was created in BioRender (Wörsdörfer, P. (2025) https://BioRender.com/rz8x29g). **(B)** Semiquantitative RT-PCR detecting mesodermal (*TBXT*, *KDR*, *GATA4*, *RUNX1*), cardiac (*NKX2.5*, *TBX5*, *ISL1*, *TNNT2*, *DES*, *RYR2*), and endothelial (*PECAM1*) marker gene expression at days 2, 4 and 8 of differentiation. **(C)** H&E staining of an organoid section at day 2. **(D,D′)** Immunofluorescence analysis of the organoid shown in C detecting early mesodermal markers TBXT and VIM at day 2 of organoid development. **(E)** Immunofluorescence analysis of the organoid shown in C detecting early mesodermal markers GATA6 and KDR at day 2 of organoid development. **(F)** H&E staining showing hollow cavity-like structures in cardiac organoids at day 7. **(G,G′)** Immunofluorescence analysis of the organoid shown in F detecting mesenchymal cells (VIM^+^) in close association with cardiomyocytes (TNNT2^+^) at day 7 of organoid development. **(H)** H&E staining of organoid sections at day 35 of development. **(I)** Immunofluorescence analysis of the organoid shown in H showing close interaction of fibroblasts (VIM^+^) and cardiomyocytes (TNNT2^+^) at day 35 of organoid development. **(J)** Cardiac organoids progressively increase in size (day 0 = 712.2 ± 6.8 µm, n = 72; day 7 = 1,231 ± 20.3 µm, n = 70; day 25 = 1,613 ± 45.4 µm, n = 41. ***p < 0.001, ****p < 0.0001. **(K)** Contraction frequency of organoids increases over time, from 25 ± 1.9 bpm at day 7–57 ± 10.5 bpm at day 35 (n = 8). **p < 0.01, ****p < 0.0001.

A schematic overview of the protocol timeline is provided in Figure 1A.

### Dissociation of organoids for scRNAseq analysis

48 organoids were pooled and dissociated using a cold-activated protease protocol. The dissociation solution consisted of 10 mg/mL *Bacillus licheniformis* protease (Creative Enzymes, NATE-0633) and 125 U/mL DNase I (Merck, DN25) in ice-cold DPBS (Sigma-Aldrich) supplemented with 5 mM CaCl2 (Carl Roth), prepared in a total volume of 1 mL.

Organoids were washed once with ice-cold PBS containing 10% fetal calf serum (FCS) (Biowest). After discarding the washing solution, 1 mL of the dissociation solution was added. Organoids were incubated on ice for 15 min, during which they were gently triturated every minute using a P1000 pipette.

The dissociation reaction was stopped by adding 1 mL of PBS with 10% FCS, followed by centrifugation at 300 × g for 3 min. The resulting cell pellet was resuspended in 10 mL of PBS with 10% FCS and filtered through a pre-wetted 40 μm cell strainer.

Single cells were counted using a Neubauer hemocytometer, then centrifuged again at 300 × g for 3 min. The supernatant was discarded, and the cell pellet was resuspended in PBS containing 0.04% bovine serum albumin (BSA) for scRNA sequencing analysis.

### Single-cell RNA-Sequencing

Library preparation was performed according to the manufacturer’s instructions (Chromium Next GEM Single Cell 3′ v3.1 protocol CG000390 Rev B, 10x Genomics). Briefly, cells were resuspended in the master mix and loaded, along with partitioning oil and gel beads, into the microfluidic chip to generate a gel beademulsion (GEM). The poly-A RNA from the cell lysate contained within each droplet was reverse transcribed into cDNA, which included an Illumina R1 primer sequence, a Unique Molecular Identifier (UMI), and the 10x Barcode.

The pooled, barcoded cDNA was then purified using Silane DynaBeads, amplified by PCR, and size-selected using SPRIselect reagent to isolate appropriately sized fragments for subsequent library construction. During library construction, the Illumina R2 primer sequence, paired-end constructs with P5 and P7 sequences, and a sample index were added.

Library quantification and quality control were performed using a Qubit™ 4.0 Fluorometer (Thermo Fisher) and a 2100 Bioanalyzer with the High Sensitivity DNA Kit (Agilent). Sequencing was carried out on a NovaSeq 6000 sequencer (Illumina) using an S2 flow cell.

Downstream data analysis was conducted using the Loupe Browser v8 (10x Genomics).

### Immunofluorescence analyses

Cardiac organoids were fixed in 4% paraformaldehyde (PFA; Sigma-Aldrich) at 4 °C overnight, washed in phosphate-buffered saline (PBS; Sigma-Aldrich), and embedded in 1% agarose gel (Biozym). Subsequently, 5 µm paraffin sections were prepared.

For immunofluorescence staining, sections were deparaffinized, rehydrated, and subjected to antigen retrieval using 10 mM sodium citrate buffer (pH 6.0). Primary antibodies against PECAM1 (CD31) (DAKO, M0823), VIM (Invitrogen, MA5-16409), ACTN1 (Abcam, Ab68167), KDR (Miltenyi Biotec, 130-125-988), TBXT (R&D Systems, AF2085), TNNT2 (Life Technologies, MA512960), CD34 (Life Technologies, MA5-32059), AIF1 (WAKO/Fuji Film, 019-19741) and PTPRC (CD45) (Life Technologies, 14-0451-82) were diluted in NBS blocking solution and incubated overnight at 4 °C.

Secondary antibodies conjugated to Cy2, Cy3, or Cy5 fluorophores (Dianova) were diluted in PBS and applied for 1 h at room temperature. Nuclei were counterstained with DAPI (Roche).

Additionally, sections were stained with eosin (AppliChem) and hematoxylin (Chroma) for histological evaluation. Images were acquired using an Axiovert 40 CFL microscope (Carl Zeiss Microscopy GmbH), a Keyence BZ-X fluorescence microscope, and a Nikon Eclipse Ti confocal laser scanning microscope.

### Tissue clearing

Tissue clearing was performed according to a previously published protocol (Worsdorfer et al., 2020). For immunofluorescence staining of cleared cardiac organoids, primary antibodies against PECAM1 (DAKO, M0823), ACTN1 (Abcam, Ab68167), TNNT2 (Life Technologies, MA512960), and AIF1 (WAKO/Fuji Film, 019-19741) were used.

Imaging was carried out using a Nikon Eclipse Ti confocal laser scanning microscope equipped with long working distance air objectives (4x and 20x) to acquire z-stack images. Three-dimensional reconstruction of the acquired image stacks was performed using Fiji (ImageJ) or Nikon NIS-Elements Confocal software.

### Transmission electron microscopy

Cardiac organoids were washed with PBS and kept on ice for 15 min prior to fixation. Organoids were fixed overnight at 4 °C in a solution containing 2.5% glutaraldehyde, 4% paraformaldehyde (PFA), and 2 mM CaCl_2_ in 0.1 M cacodylate buffer (composed of 50 mM cacodylate, 50 mM KCl, and 2.5 mM MgCl_2_, pH 7.2). Following fixation, samples were washed with 0.1 M cacodylate buffer and subjected to a second fixation step using 1% osmium tetroxide in 0.1 M cacodylate buffer for 1 h.

After osmium fixation, organoids were washed for 10 min in 0.1 M cacodylate buffer, followed by two 10-min washes in double-distilled water (ddH_2_O). Samples were then incubated in 8% uranyl acetate substitute for 1 h and washed again twice for 10 min in ddH_2_O.

Dehydration was carried out using a graded ethanol series (30%, 50%, 70%, 90%, and two changes of 100% ethanol), with each step lasting 10 min. Organoids were then incubated in propylene oxide (PO) for two 30-min intervals, followed by overnight incubation in a 1:1 mixture of PO and Epon 812 resin.

The following day, samples were incubated in pure Epon for 2 h and embedded by polymerizing the resin at 60 °C for 48 h. Ultrathin sections were prepared using an ultramicrotome, collected on nickel grids, and post-stained with 2.5% uranyl acetate and 0.2X lead citrate. Finally, specimens were analyzed using a LEO AB 912 transmission electron microscope (Zeiss).

### Phagocytosis assay

Four organoids were pooled and dissociated with the STEMdiff™ Cardiomyocyte Dissociation Kit (STEMCELL Technologies), and the cells were replated in 500 µL STEMdiff™Cardiomyocyte Support Medium (STEMCELL Technologies) onto Matrigel-coated coverslips. Dissociated cells were incubated with 0.05% fluorescent latex beads (Sigma) for an additional 24 h, followed by fixation in 4% PFA. Immunofluorescence analysis was performed using primary antibodies against AIF1 and CD68 to identify macrophages.

### Semiquantitative RT polymerase chain reaction

For polymerase chain reaction (PCR), samples were disrupted using ultrasonic sonication (Ultrasound Processor, 20 kHz, 80 W; power setting 30, 3 × 20 s pulses with 3 s intervals), followed by RNA extraction using the Direct-zol RNA MiniPrep Plus Kit (Zymo Research). cDNA synthesis was performed using the GoScript™ Reverse Transcriptase (Promega), following the manufacturer’s instructions. PCR amplification was carried out using the Red MasterMix (2×) Taq PCR MasterMix (Genaxxon). The following primer pairs were used:

GAPDH

Forward: TGACAACTTTGGTATCGTGGA

Reverse: CCAGTAGAGGCAGGGATGAT

NKX2.5

Forward: CCAAGGACCCTAGAGCCGAA

Reverse: ATAGGCGGGGTAGGCGTTAT

TNNT2

Forward: GGAGGAGTCCAAACCAAAGCC

Reverse: TCAAAGTCCACTCTCTCTCCATC

DES

Forward: TCGGCTCTAAGGGCTCCTC

Reverse: CGTGGTCAGAAACTCCTGGTT

ISL1

Forward: GCGGAGTGTAATCAGTATTTGGA

Reverse: GCATTTGATCCCGTACAACCT

KDR

Forward: GGCCCAATAATCAGAGTGGCA

Reverse: CCAGTGTCATTTCCGATCACTTT

PECAM1

Forward: AACAGTGTTGACATGAAGAGCC

Reverse: TGTAAAACAGCACGTCATCCTT

TBXT

Forward: TATGAGCCTCGAATCCACATAGT

Reverse: CCTCGTTCTGATAAGCAGTCAC

GATA4

Forward: CGACACCCCAATCTCGATATG

Reverse: GTTGCACAGATAGTGACCCGT

TBX5

Forward: CTGTGGCTAAAATTCCACGAAGT

Reverse: GTGATCGTCGGCAGGTACAAT

RYR2

Forward: ACAACAGAAGCTATGCTTGGC

Reverse: GAGGAGTGTTCGATGACCACC

RUNX1

Forward: CTGCCCATCGCTTTCAAGGT

Reverse: GCCGAGTAGTTTTCATCATTGCC

### Calcium imaging

For calcium imaging, organoids were incubated with 1 µM Fluo-4 AM (Gibco) in DMEM (Gibco) at 37 °C for 30 min. For stimulation experiments, the Fluo-4 AM-containing medium was replaced with DMEM supplemented with 100 µM isoproterenol (Sigma, dissolved in ethanol) and incubated at 37 °C for an additional 30 min, followed by a final medium change.

Dynamic fluorescence changes were recorded before and after treatment using a Leica DM IL LED microscope equipped with a Leica EL6000 external fluorescence light source and a Leica DFC450C camera. Videos were processed using Leica Application Suite Version 4.12.0. Fluorescence data were analyzed using Fiji (ImageJ) and Microsoft Excel.

### Statistics

All statistical analyses were performed using GraphPad Prism version 10. Data are presented as bar graphs showing mean ± standard error of the mean (SEM), with asterisks indicating statistical significance where applicable.

Normality of data distribution was assessed using the Shapiro–Wilk test. For normally distributed data, statistical significance was determined using one-way ANOVA followed by Bonferroni’s multiple comparison test or Kruskal-Wallis test with Dunn`s multiple-comparison test.

## Results

### Generation of organoids

To generate the organoids, modifications were made to a previously published protocol for self-assembling cardiac organoids (Lewis-Israeli et al., 2021). In contrast to the published method, the WNT antagonist IWP2 was used instead of WNT-C59, and the organoids were cultured on an orbital shaker. Of note, IWP2 was used at a low concentration of 2 µM. Finally, the second supplementation with CHIR99021, which was used in the original protocol to induce epicardial cells, was omitted (Figure 1A).

Initial characterizations using semiquantitative RT-PCR, demonstrated that the expression of marker genes for mesoderm specification (*TBXT, KDR, GATA4, RUNX1*), heart development (*NKX2.5, TBX5, ISL1*), cardiomyocytes (*TNNT2, DES, RYR2*), and endothelial cells (*PECAM1, RUNX1*) became upregulated over the course of differentiation. While mesodermal markers were detectable at day 2, cardiac and vascular transcripts were first detected at day 8 (Figure 1B).

To confirm the RT-PCR findings, we performed histological analyses. H&E staining on paraffin sections from organoids at day 2 of development revealed a uniform and cell-rich mesenchymal tissue (Figure 1C; Supplementary Figure S1A). Immunofluorescence staining on these sections demonstrated the expression of the mesodermal transcription factor TBXT (Figure 1D). Moreover, expression of the mesenchymal marker VIM was detectable (Figure 1D). Furthermore, we observed GATA6 expression and expression of the VEGF receptor 2 (KDR), typical hallmarks of mesoderm formation (Figure 1E). In day 8 organoids (Figure 1F; Supplementary Figure S1B), the tissue was loosely arranged with large cavities and a core of epithelial cells. Cells expressing the mesenchymal/fibroblast marker VIM were detected in close association with cells expressing the cardiomyocyte marker TNNT2. Both were located in the periphery of the organoid (Figures 1F,G). At day 35, the tissue architecture of the organoid became more compact (Figure 1H). The organoid showed areas of myocardial tissue formed by TNNT2^+^ cardiomyocytes and VIM^+^ fibroblasts (Figure 1I). In addition, epithelial structures and loose connective tissue co-developed (Supplementary Figure S1D). During differentiation, organoids significantly increased in size from a diameter of approximately 700 µm (712.2 ± 6.8 µm, n = 72) at day 0 to a diameter of 1,600 μm at day 25 (1,600 ± 45 μm, n = 41) (Figure 1J). Spontaneous contractions started between day 7 and day 10, and contraction frequencies increased significantly from initially 25 ± 1.9 (n = 9) to 57 ± 10.5 (n = 9) contractions per minute after 4 weeks in culture (Figure 1K).

### Histological characterization of cardiomyocytes

**FIGURE 2:**
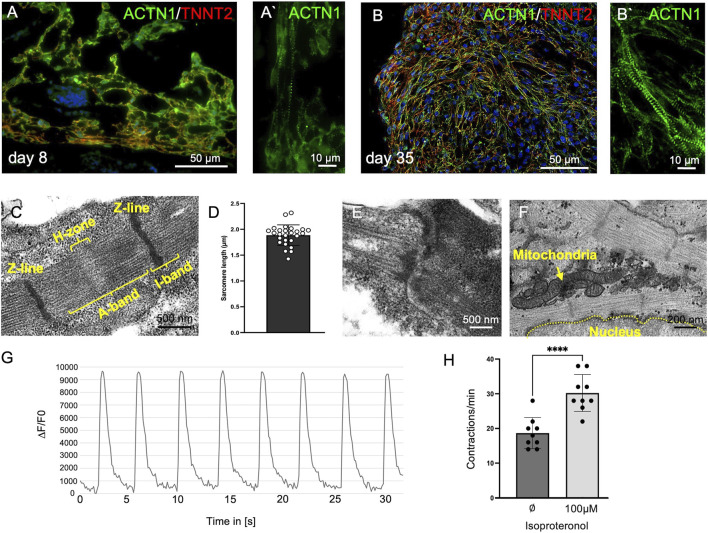
Cardiomyocyte Maturation in Organoids. **(A)** Immature cardiomyocytes in day 8 organoids. **(A′)** Few sarcomere-like structures are visible, indicated by a sparse cross-striated staining pattern of ACTN1/TNNT2 with a limited number of repeats. **(B)** Cardiac organoids at day 35. **(B′)** Highly repetitive and well-developed cross-striated staining pattern of ACTN1 and TNNT2, indicating cardiomyocyte maturation. **(C)** Transmission electron microscopy reveals sarcomeres at day 35, exhibiting clear banding with repetitive Z-lines, distinct I-bands, A-bands, and an H-zone. **(D)** Sarcomere length measurements show an average length of 1.9 µm (±0.2 µm; n = 26). **(E)** Transmission electron microscopy reveals intercalated disc-like structures. **(F)** Transmission electron microscopy shows multiple mitochondria adjacent to sarcomeres. **(G)** Cardiomyocytes within the organoids exhibit regular calcium transients synchronized with spontaneous contractions at day 35 of organoid development. **(H)** Cardiomyocytes increase their contraction frequency from approximately 18 ± 4.47 bpm to 30 ± 5.33 bpm in response to treatment with 100 µM isoproterenol (n = 9; ****: *p* < 0.0001) at day 25 of organoid development.

In day 8 organoids, ACTN1 and TNNT2 exhibited a diffuse staining pattern, with only a few immature, sarcomere-like structures visible. By contrast, at day 35, a continuous cross-striated staining pattern emerged, indicating a higher organized actin-myosin arrangement (Figures 2A,B). Electron microscopic analyses confirmed the formation of well-organized sarcomeres with clearly visible Z-lines, as well as A- and I-bands and the H-zone. Sarcomeres at day 35 showed a length of approximately 1.9 µm (1.89 ± 0.2 µm; n = 26) (Figures 2C,D). In addition, intercalated disc-like structures were observed between neighboring cardiomyocytes (Figure 2E). Multiple mitochondria were found near the sarcomeres (Figure 2F). Moreover, cardiomyocytes exhibited regular calcium transients accompanying each contraction (Figure 2G). The stimulation with 100 µM isoproterenol clearly increased the beating frequency (untreated: 18 ± 4.5 bpm, treated: 30 ± 5.3 bpm; n = 9; p=<0.0001), demonstrating a response of the cardiac tissue to beta-adrenergic stimulation (Figure 2H).

### Histological characterization of endothelial cells

**FIGURE 3:**
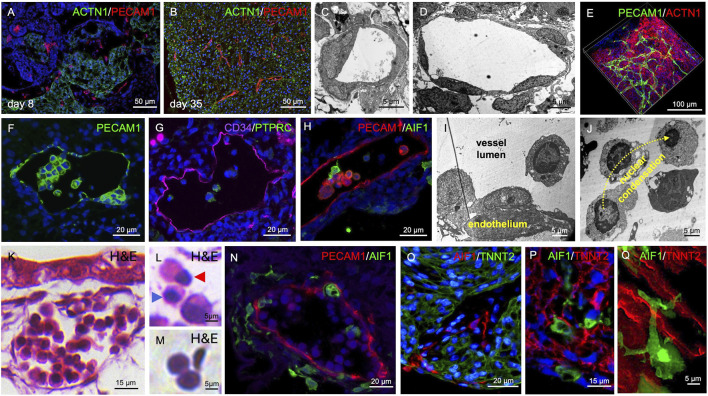
Vasculogenesis and Hematopoiesis in Cardiac Organoids. **(A)** PECAM1^+^ cell islands and endothelial cords are detectable in day 8 organoids. **(B)** By day 35, PECAM1^+^ cells form endothelial cords and capillary-like structures within cardiac tissue (ACTN1^+^). **(C,D)** Transmission electron microscopy reveals capillary-like structures with smaller **(C)** and larger **(D)** luminal diameters at day 35 of organoid development. **(E)** Whole-mount tissue clearing shows capillary-like structures and endothelial cords forming a branched network within the myocardial tissue at day 35 of organoid development. **(F)** PECAM1^+^ intraluminal clusters are observed within endothelial-lined cavities at day 35 of organoid development. **(G)** Intraluminal cells express CD34 and PTPRC at day 35 of organoid development. **(H)** AIF1-expressing cells are also present within the endothelial-lined cavities at day 35 of organoid development. **(I)** Transmission electron microscopy reveals hemogenic endothelium-like structures at day 35 of organoid development. **(J)** Intraluminal cells exhibit chromatin condensation reminiscent of erythropoiesis at day 35 of organoid development. **(K–M)** H&E staining shows vessel-like structures containing intraluminal hematopoietic cells at day 35 of organoid development. Different stages of erythropoiesis **(L)** and biconvex, disc-shaped erythrocyte-like cells **(M)** are detectable. Blue arrowhead in **(L)** indicates a cell with a condensed nucleus; red arrowhead indicates a cell undergoing nuclear extrusion. **(N)** AIF1^+^ cells are found within and surrounding vessel-like structures at day 35 of organoid development. **(O,P)** AIF1^+^ macrophages are also present within the musculature of cardiac organoids at day 35 of organoid development. **(Q)** High-magnification imaging reveals close interactions between AIF1^+^ macrophages and TNNT2^+^ cardiomyocytes at day 35 of organoid development.

We also detected endothelial cells in the organoids. At day 8, PECAM1 staining revealed primitive endothelial cords between patches of ACTN1^+^ cardiac tissue (Figure 3A). At day 35, PECAM1^+^ cells formed a network of endothelial cords throughout the cardiac tissue, and capillary-like structures with a clearly visible lumen were detected (Figure 3B). Transmission electron microscopy confirmed lumen formation and demonstrated the presence of larger and smaller capillary-like structures (Figures 3C,D). Moreover, tissue clearing analysis showed the development of a continuous and branched endothelial network (PECAM1^+^) within the cardiac muscle (ACTN1^+^) (Figure 3E, Supplementary Video S1).

### Histological characterization of hematopoietic cells including macrophages

PECAM1^+^ cells were not only detected lining larger cavities but were also found within their lumens (Figure 3F). Besides PECAM1, the endothelial cells also expressed CD34 and a fraction of cells within the vascular lumen were PTPRC^+^, suggesting hematopoiesis from hemogenic endothelium (Figure 3G). Moreover, AIF1^+^ cells could be detected in the vessel lumen indicating differentiation towards the macrophage lineage (Figure 3H). Electron microscopic images showed intra-luminal endothelial clusters (Figure 3I) and free floating intra-luminal cells exhibiting a gradual condensation of their nucleus as observed during erythropoiesis (Figure 3J). H&E staining revealed vessels filled with hematopoietic cells, reminiscent of vessels in the early embryo. (Figure 3K, Supplementary Video S2). We detected cells showing characteristics of different stages of erythropoiesis, such as nuclear condensation and extrusion (Figure 3L) as well as biconvex, disc-shaped erythrocyte-like cells (Figure 3M). AIF1^+^ macrophage-like cells were found both within the vascular lumen and in the connective tissue surrounding the PECAM1^+^ vessels (Figure 3N, Supplementary Video S3). The macrophages appeared to spread from the vessels into all regions of the organoid and became also present within the cardiac tissue (Figure 3O; Supplementary Figure S1C, Supplementary Video S4), getting in close contact with TNNT2^+^ cardiomyocytes (Figures 3P,Q, Supplementary Video S5).

### Single Cell RNA sequencing (scRNAseq) analysis of organoids

**FIGURE 4:**
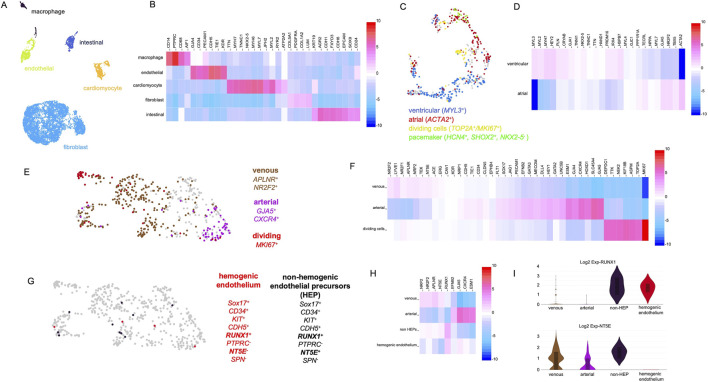
Single-cell RNA-sequencing analysis of organoids at day 35 of development. **(A)** Single-cell RNA-sequencing reveals distinct cell clusters characterized by gene expression profiles typical of fibroblasts, cardiomyocytes, endothelial cells, macrophages, and intestinal epithelial cells. **(B)** Heatmap showing marker genes used to identify the clusters depicted in panel **(A)**. **(C)** Detailed analysis of the cardiomyocyte cluster reveals cells with atrial, ventricular, and pacemaker cell-like identities. **(D)** Heatmap showing genes differentially expressed between the cardiomyocyte subpopulations shown in panel **(C)**. **(E)** Detailed analysis of the endothelial cell cluster reveals cells with arterial (purple) and venous (brown) identities, as well as proliferating cells (red). **(F)** Heatmap showing genes differentially expressed between the endothelial subpopulations shown in panel **(E)**. **(G)** Further analysis of the endothelial cluster reveals cells with gene expression patterns reminiscent of hemogenic endothelial cells (red) and non-hemogenic endothelial progenitor cells (black). **(H)** Heatmap showing genes differentially expressed between the subpopulations shown in panel **(G)**. **(I)** Violin plots showing the expression of *RUNX1* and *NT5E* across the cell populations depicted in panels **(E,G)**.

Day 35 organoids were dissociated, and single-cell RNA sequencing was performed. The analysis revealed that organoids contained fibroblast-like cells, cardiomyocytes, endothelial cells, and macrophages (Figure 4A). In addition, cells with an expression profile of intestinal epithelial cells were observed (Figures 4A,B). Intestinal epithelial structures were also visible in histological sections of organoids (Supplementary Figure S1D). Figure 4B shows a heatmap of selected genes, which served as the basis for assigning the clusters to their cellular identities.

After this initial characterization, the individual cell clusters were analyzed in more detail. First, we focused on the cardiomyocyte population and investigated the expression of markers for atrial and ventricular cardiomyocytes utilizing a panel of marker genes previously described for a cardiac organoid model (Volmert et al., 2023). Ventricular (*MYL3* ^*+*^) as well as atrial cardiomyocytes (*ACTA2* ^*+*^) were detected. Moreover, a cluster of actively dividing cardiomyocytes (*MKI67* ^*+*^) as well as a cluster of cells expressing genes in a pattern characteristic for pacemaker cells (*HCN4* ^*+*^ *,SHOX2* ^*+*^ *,NKX2-5* ^−^) were observed (Figure 4C). A detailed heatmap of all marker genes tested is depicted in Figure 4D.

Next, the endothelial cluster was analyzed in detail. The cluster contained 510 cells out of which 345 were PECAM1^+^/CD34^+^ and 165 were PECAM1^+^/CD34^-^ which suggests that some endothelial cells were actively participating in angiogenesis (*CD34* ^*+*^), while others had already integrated into capillary-like structures. A subset of endothelial cells expressed elevated levels of venous (*APLNR* and *NR2F2*) and another subset arterial marker genes (*GJA5*, *CXCR4*, and *HEY2*) (Figure 4E). Additionally, a cluster of *MKI67* ^+^ cells was identified, indicating actively dividing endothelial cells. Taken together, this expression data indicates the emergence of vascular specialization within an immature and developing vascular network. Figure 4F presents a heatmap showing the expression levels of arterial, venous, and cell cycle-associated marker genes.

Based on our histological analyses, we assumed that the observed hematopoietic cells (macrophages, erythrocytes) originate from hemogenic endothelium. To strengthen this hypothesis, the endothelial cell cluster was examined for cells with a characteristic gene expression profile of hemogenic endothelial cells. iPSC-derived hemogenic endothelial cells have been shown to express *CD34*, *CDH5*, *SOX17*, *KIT*, and the hematopoietic master regulator *RUNX1* (Choi et al., 2012). Moreover, these cells are typically negative for *PTPRC*, *NT5E* (*CD73*), and *SPN*. A related population of non-hemogenic endothelial precursors with a similar gene expression profile has also been described, distinguishable by their expression of *NT5E* (*CD73*) (Choi et al., 2012). We indeed detected rare cells matching these expression profiles in the organoids endothelial cell population (Figure 4G). Figure 4H presents a heatmap demonstrating that the hemogenic endothelium-like cells expressed a combination of arterial and venous markers, displayed *RUNX1* expression, and lacked *NT5E* (*CD73*) expression, consistent with a previous report (Choi et al., 2012). The violin plots in Figure 4I compare the expression levels of *RUNX1 and NT5E* across the endothelial subpopulations.

**FIGURE 5:**
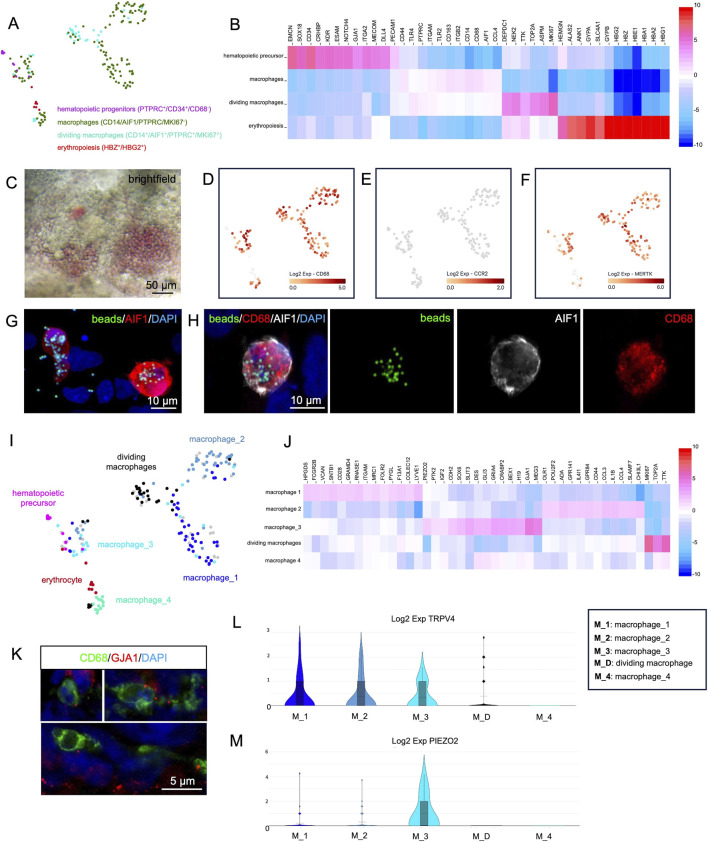
Single-cell RNA-sequencing analysis of hematopoietic cells derived from cardiac organoids at day 35 of development. **(A)** Reanalysis of cells exhibiting characteristic gene expression profiles of hematopoietic progenitors, macrophages, dividing macrophages, and erythropoietic cells. **(B)** Heatmap showing genes differentially expressed between the clusters shown in panel **(A)**. **(C)** Brightfield microscopy of living organoids reveals the red color of hemoglobin produced in erythrocytes. **(D–F)** Expression of *CD68* **(D)**, *CCR2* **(E)**, and *MERTK* **(F)** in the reanalyzed cells from panel **(A)**. **(G,H)** Macrophages (CD68^+^/AIF^+^) isolated from dissociated organoids phagocytose fluorescently labeled latex beads (green). Nuclei are counterstained with DAPI. **(I)** Detailed analysis of the cell clusters from panel A identifies four macrophage subclusters: *macrophage_1*, *macrophage_2*, *macrophage_3*, and *macrophage_4*. **(J)** Heatmap showing genes differentially expressed between the macrophage subclusters shown in panel **(I)**. **(K)** A subset of macrophages (CD68^+^) within the organoids expresses GJA1. Nuclei are counterstained with DAPI. **(L,M)** Violin plots showing the expression of *TRPV4* **(L)** and *PIEZO2* **(M)** across the macrophage populations identified in panel **(I)**.

To further characterize the organoids hematopoietic cell types, we focused on three distinct populations: cells expressing hematopoietic progenitor markers (*CD34* ^*+*^, *PTPRC* ^*+*^, *CD68* ^*−*^), fetal erythrocyte markers (*HBZ* ^*+*^, *HBG1* ^*+*^), and macrophage markers (*CD14* ^*+*^, *AIF1* ^*+*^, *PTPRC* ^*+*^). Additionally, we distinguished between proliferating and non-proliferating macrophages based on *MKI67* expression. Cells with these characteristics were extracted from the original dataset and reanalyzed (Figure 5A). Figure 5B presents a heatmap showing additional marker genes for hematopoietic progenitors, dividing and non-dividing macrophages, as well as erythrocytes.

The erythrocyte population expressed fetal hemoglobin genes (*HBG2* ^*+*^ *, HBZ* ^*+*^ *, HBG2* ^*+*^ *, HBE* ^*+*^) (Figure 5B) and lacked expression of the adult *HBB*. In some organoids, the production of hemoglobin could be directly observed by the red color of densely packed erythrocytes within endothelium-lined cavities (Figure 5C).

All detected macrophages expressed *CD68* (Figure 5D) but were negative for *CCR2* (Figure 5E), a hallmark of tissue-resident macrophages of embryonic origin (Bajpai et al., 2018).

### Macrophages are capable of phagocytosis

Resident macrophages *in vivo* contribute to cardiac function by MERTK dependent phagocytosis of exopheres, e.g., to remove non-functional mitochondria (Nicolas-Avila et al., 2020). Therefore, *MERTK* expression was checked in organoid macrophages and was detected in most cells (Figure 5F). To test the phagocytic capability of organoid-derived macrophages, a phagocytosis assay with fluorescent latex beads was performed on dissociated organoids. Multiple AIF1^+^/CD68^+^ macrophages that had internalized fluorescent beads were detected, while non-macrophage cells rarely showed intracellular beads (Figures 5G,H).

### Analysis of macrophage subclusters in organoids

A more detailed analysis of the organoid macrophages revealed at least four distinct subclusters, which we termed “macrophage_1”, “macrophage_2”, “macrophage_3” and “macrophage_4” (Figure 5I). The macrophage_1 cluster showed high expression of *LYVE1*, *ITGAM* and *SIGLEC1*, the macrophage_2 cluster was characterized by expression of *ADGRE1* and *SLAMF7* and the macrophage_3 cluster showed high expression of *GJA1*, *MEG3* and *IGF2*. In contrast, the macrophage_4 cluster did not display any specific marker genes beyond the general pan-macrophage markers. In addition, a cluster of dividing macrophages was identified by *MKI67* expression (Figure 5J). Of particular interest was the macrophage_3 cluster. It has been reported that, cardiomyocytes and macrophages in the mouse directly interact via gap junctions composed of GJA1 (Hulsmans et al., 2017) and that TRPV4-expressing macrophages can be activated by mechanical stretch, leading to the release of growth factors that contribute to cardiac remodeling (Wong et al., 2021). Cluster macrophage_3 strongly expressed *GJA1* along with additional genes involved in cell-cell junction formation (*CDH2*, *DES*) and focal adhesion contacts (*PTK2*).

The expression of GJA1 protein in a subset of organoid macrophages could be verified by immunofluorescence analysis, revealing a gap junction plaque-like punctate staining pattern at the macrophage surface (Figure 5K). A subset of cells across all macrophage clusters expressed *TRPV4* (Figure 5L). In addition, the macrophage_3 cluster showed elevated expression of *PIEZO2*, a mRNA encoding the respective mechanosensor (Figure 5M) as well as *IGF2*, a growth factor which is known to play important roles in cardiac development and regeneration (Li et al., 2011; Leid et al., 2016; Shen et al., 2020) (Figure 5J).

### Comparison of organoid macrophages to macrophages in human fetal heart tissue

To assess whether organoid macrophages are comparable to macrophages in the developing heart, we compared our scRNA-seq data to previously published scRNA-seq data from human hearts obtained from an 83-day-old fetus (Miao et al., 2020), accessible via the Single Cell Portal (https://singlecell.broadinstitute.org/single_cell). The dataset includes an immune cell cluster (Supplementary Figure S3A), within which 138 cells express *CD14, AIF1*, and *CD68*, and are *CCR2* ^-^ identifying them as tissue resident macrophages.

Among these 138 fetal cells:

- 37 (27%) co-expressed the marker genes *LYVE1*, *HPGDS*, and *ITGAM*, which are highly expressed in the organoid macrophage cluster macrophage_1 (Supplementary Figure S3B).
- 18 (13%) showed co-expression of *GJA1*, *MEG3*, and *IGF2*, genes highly expressed in the organoid macrophage cluster macrophage_3 (Supplementary Figure S3B).
- No cells co-expressed *ADGRE1*, *SLAMF7*, and *IL4I1* as found in the organoid macrophage cluster macrophage_2. In general, *ADGRE1*-expressing macrophages were absent from the fetal dataset, and only 3 macrophages expressed *SLAMF7* (Supplementary Figure S3B).

The cluster macrophage_4 was not compared to the fetal dataset, as it lacks specific marker genes clearly distinguishing it from the other clusters, which hindered a meaningful comparison.

The initial comparison demonstrated that the organoid macrophage clusters macrophage_1 and macrophage_3 show similarities to tissue-resident macrophages found in the fetal heart. Therefore, we decided to analyze them in more detail. These organoid macrophage clusters could be clearly distinguished by their high expression of *LYVE1* or the presence of *GJA1*, respectively (Supplementary Figure S3C).

To this end, we sorted the fetal dataset to identify the 25 cells within the immune cell cluster with the highest *LYVE1* expression and the 25 with the highest *GJA1* expression.

In these two groups of selected fetal cells, we examined the expression of characteristic marker genes found in the organoid macrophage clusters macrophage_1 and macrophage_3 (Supplementary Figure S3D). The comparison showed that the fetal *GJA1*-high and *LYVE1*-high tissue-resident macrophages exhibit a high degree of similarity to the cells in the organoid clusters macrophage_3 and macrophage_1, respectively, with few exceptions such as *SLIT3* or *VCAN* expression (Supplementary Figure S3E).

## Discussion

Here, we describe organoids generated from human iPSCs that contain mesenchymal and myocardial tissue areas as well as intestinal epithelium. Within the myocardial areas, cardiomyocytes undergo progressive maturation over time, developing well-organized sarcomeres and show an increase in spontaneous rhythmic contractions. The myocardial tissue is interspersed by fibroblasts and contains an endothelial network with artery- and vein-like endothelial domains. Endothelium-lined cavities within the organoid contain hemogenic endothelial cells that have blood-forming properties. The hemogenic endothelium gives rise to hematopoietic clusters inside the cavity lumen, which generate erythrocytes and *CCR2* ^*-*^ tissue-resident macrophages (Bajpai et al., 2018). The macrophages infiltrate the myocardial tissue, coming into close contact with cardiomyocytes.

Our method was inspired by a previously published cardiac organoid protocol (Lewis-Israeli et al., 2021). However, we replaced the WNT antagonist WNT-C59 by IWP2 and applied it at a low concentration of 2 µM. Under these conditions we observed the appearance of macrophages and other hematopoietic cells which have been explicitly reported to be absent using the original protocol (Lewis-Israeli et al., 2021; Kostina et al., 2023; Volmert et al., 2023; O'Hern et al., 2024). We hypothesize that the low dose of IWP2 (2 µM) leads to less tight and less efficient WNT inhibition, making the culture more permissive for the development of hematopoietic cells and other tissue types such as intestinal epithelium alongside the cardiomyocyte lineage. Notably, if IWP2 is used for cardiac organoid differentiation, it is usually applied at higher concentration of 5 µM (Drakhlis et al., 2021). IWP2 and WNT-C59 are both PORCN inhibitors (Shah et al., 2021), however, IWP2 was previously used for the specification of primitive hematopoietic cells from human iPSCs (Sturgeon et al., 2014) and was therefore favored.

The finding that iPSC-derived mesodermal organoids have the potential to give rise to cells of the hematopoietic lineage is not new. For example, our lab could demonstrate that vascularized mesodermal organoids give rise to microglia-like cells when co-cultured with neural organoids (Worsdorfer et al., 2019). Other studies show that this capacity can be further enhanced by supplementing the cultures with hematopoietic growth factors such as SCF, IL-3, IL-6, TPO, and M-CSF, resulting in the formation of blood-producing bone marrow organoids (Frenz-Wiessner et al., 2024; Olijnik et al., 2024). A recent report further showed that applying a precisely timed sequence of four complex growth factor cocktails to cardiac organoids can induce CD45^+^ hematopoietic cells (Dardano et al., 2024). Here, we show that hematopoiesis and macrophage formation can innately occur without adding additional growth factors, when low concentrations of IWP2 are used. Nevertheless, incorporating hematopoietic growth factors into our easy and cost-efficient protocol may offer opportunities for further optimization and will be tested in future experiments.

Recent studies in mice suggest that macrophages closely interact with cardiomyocytes, either directly through gap junctional communication or indirectly by growth factor release (Hulsmans et al., 2017; Wong et al., 2021). Macrophages that connect to the extracellular matrix via focal adhesion complexes respond to mechanical stretch via a TRPV4-mediated signaling pathway, which controls growth factor expression and growth factor-induced cardiac remodeling. Gap junctions between macrophages and cardiomyocytes play a role in facilitating electrical conduction. Moreover, cardiac macrophages help remove defective mitochondria from cardiomyocytes through exosphere phagocytosis. Depletion of macrophages or defects in the phagocytic receptor MERTK lead to impaired autophagy, accumulation of defective mitochondria, and consequently, metabolic changes and heart dysfunction (Nicolas-Avila et al., 2020). All these findings show that tissue-resident macrophages play an important role in maintaining functional cardiac tissue and are probably also involved in cardiac development and tissue maturation. This assumption is supported by recent studies showing that adding macrophages to engineered heart tissue improves contractile strength, functionality, and long-term vascularization (Hamidzada et al., 2024; Landau et al., 2024; Lock et al., 2024).

While our data do not allow us to proof whether the observed organoid-derived macrophages perform functions equivalent to their *in vivo* counterparts, we at least provide evidence of their phagocytic activity and transcriptional similarity to macrophages found in fetal heart tissue. Of special interest is the expression of genes associated with the biological processes described above, including *GJA1, MERTK, IGF2*, and *VEGFA*. TRPV4 mRNA was also detected, and we additionally identified expression of another mechanosensor, PIEZO2, typically associated with nerve cells (Coste and Delmas, 2024), particularly in GJA1^+^ macrophages. This finding aligns with gene expression data from macrophages in fetal heart tissue.

Future studies are required to systematically investigate the impact of organoid macrophages on cardiac tissue development, maturation and function. These will include macrophage depletion experiments and comprehensive characterization of the organoid myocardium.

In conclusion, we present a novel organoid model in which tissue-resident macrophages, an important cardiac cell type previously absent from existing cardiac organoid systems, innately co-develop with and integrate into the myocardium.

## Data Availability

The datasets presented in this study can be found in online repositories. The names of the repository/repositories and accession number(s) can be found below: https://www.ncbi.nlm.nih.gov/geo/, GSE307256.

## Supplementary material

SUPPLEMENTARY FIGURE S1 Reproducibility of Organoid Formation. **(A)** H&E staining of eight randomly selected organoids at day 2 of development from the same differentiation batch, embedded in a single agarose block and sectioned as one specimen. **(B)** H&E staining of eight randomly organoids at day 8 of development from the same differentiation batch, embedded in a single agarose block and sectioned as one specimen. **(C)** Immunofluorescence analysis of eight randomly selected organoids at day 35 of development from the same differentiation batch, embedded in a single agarose block and sectioned as one specimen, showing TNNT2^+^ cardiomyocytes and AIF1^+^ macrophages. Nuclei are counterstained with DAPI. **(D)** Organoids at day 35 of differentiation contain myocardium, intestinal epithelial structures, and mesenchymal tissue. TNNT2^+^ cardiomyocytes and AIF1^+^macrophages are highlighted using immunofluorescence analysis.

SUPPLEMENTARY FIGURE S2 Reproduction of the organoid protocol using KK iPSCs. **(A)** A representative organoid at day 35 of development derived from KK iPSCs is shown. TNNT2^+^ cardiomyocytes and VIM^+^ fibroblasts are detected by immunofluorescence analysis. Nuclei are counterstained with DAPI. **(B)** Higher magnification of the area indicated by the white box in panel **(A)**. **(C)** The image shows a PECAM1^+^ endothelium-lined cavity and endothelial sprouts. PECAM1^+^ hematopoietic cells are visible within the cavities lumen. AIF1^+^ macrophages are detected both within the lumen and throughout the tissue. Nuclei are counterstained with DAPI. **(D)** The same specimen and field as in panel **(C)** is shown. The endothelium-lined cavity is marked by a white dotted line. Immunofluorescence analysis highlights AIF1^+^ macrophages and TNNT2^+^ cardiomyocytes. Nuclei are counterstained with DAPI.

SUPPLEMENTARY FIGURE S3 Comparison of Organoid Macrophages with Macrophages from Human Fetal Heart. **(A)** Previously published scRNAseq data from human fetal heart tissue (day 83) (Miao et al., 2020) contain various cell populations, including a cluster of immune cells expressing the macrophage markers *CD14*, *AIF1*, and *CD68*. **(B)** This immune cell cluster includes tissue-resident macrophages (*CD14* ^*+*^ */AIF1* ^*+*^ */CD68* ^*+*^ *, CCR2* ^*-*^). Among these, 27% co-express *LYVE1*, *HPGDS*, and *ITGAM*, while 13% co-express *GJA1*, *MEG3*, and *IGF2*. Cells expressing *ADGRE1*, *SLAMF7*, and *IL4I1* are not detectable. **(C)** Violin plots demonstrate that *GJA1* and *LYVE1* expression effectively distinguish between the macrophage populations *macrophage_1* and *macrophage_3* identified in the organoids. **(D)** The heatmap shows genes differentially expressed between *macrophage_1* and *macrophage_3*. These genes are also used in panel E to identify corresponding macrophage populations in the fetal heart tissue. **(E)** The 25 tissue-resident macrophages with the highest *LYVE1* expression and the 25 with the highest *GJA1* expression from the fetal heart scRNAseq dataset are analyzed for the expression of marker genes identified in the organoid macrophage populations macrophage_1 and macrophage_3.

SUPPLEMENTARY VIDEO S1 Endothelial Network within the Myocardial Tissue of an Organoid at Day 35 of Development. The video presents a 3D reconstruction of a z-stack acquired via confocal laser scanning microscopy following immunofluorescence staining for PECAM1 and TNNT2, combined with tissue clearing.

SUPPLEMENTARY VIDEO S2 Erythrocytes in Organoids. The video presents a series of bright-field recordings from three independent organoids, showing erythrocytes (round cells, red in color) within endothelium-lined cavities and capillary-like structures moved by spontaneous contractions of cardiomyocytes. Videos were recorded at day 21 of organoid development.

SUPPLEMENTARY VIDEO S3 Macrophages in Close Association with an Endothelium-lined Cavity. The video presents a 3D-reconstruction of a z-stack acquired via confocal laser scanning microscopy following immunofluorescence staining for PECAM1 and AIF1, combined with tissue clearing in organoids at day 35 of development.

SUPPLEMENTARY VIDEO S4 Macrophages within the Organoids Myocardial Tissue. The video presents a 3D reconstruction of a z-stack acquired via confocal laser scanning microscopy following immunofluorescence staining for ACTN1 and AIF1, combined with tissue clearing in organoids at day 35 of development.

SUPPLEMENTARY VIDEO S5 Macrophages within the Organoids Myocardial Tissue. The video presents a 3D reconstruction of a z-stack acquired via confocal laser scanning microscopy following immunofluorescence staining for TNNT2 and AIF1, combined with tissue clearing in organoids at day 35 of development.
