# Supplementary figures and images for: Tissue-resident macrophages co-develop with myocardial tissue in human induced pluripotent stem cell-derived organoids
Source: Front Cell Dev Biol. 2025 Nov 13;13:1629988. doi: 10.3389/fcell.2025.1629988 (PMC12657418; doi:10.3389/fcell.2025.1629988)

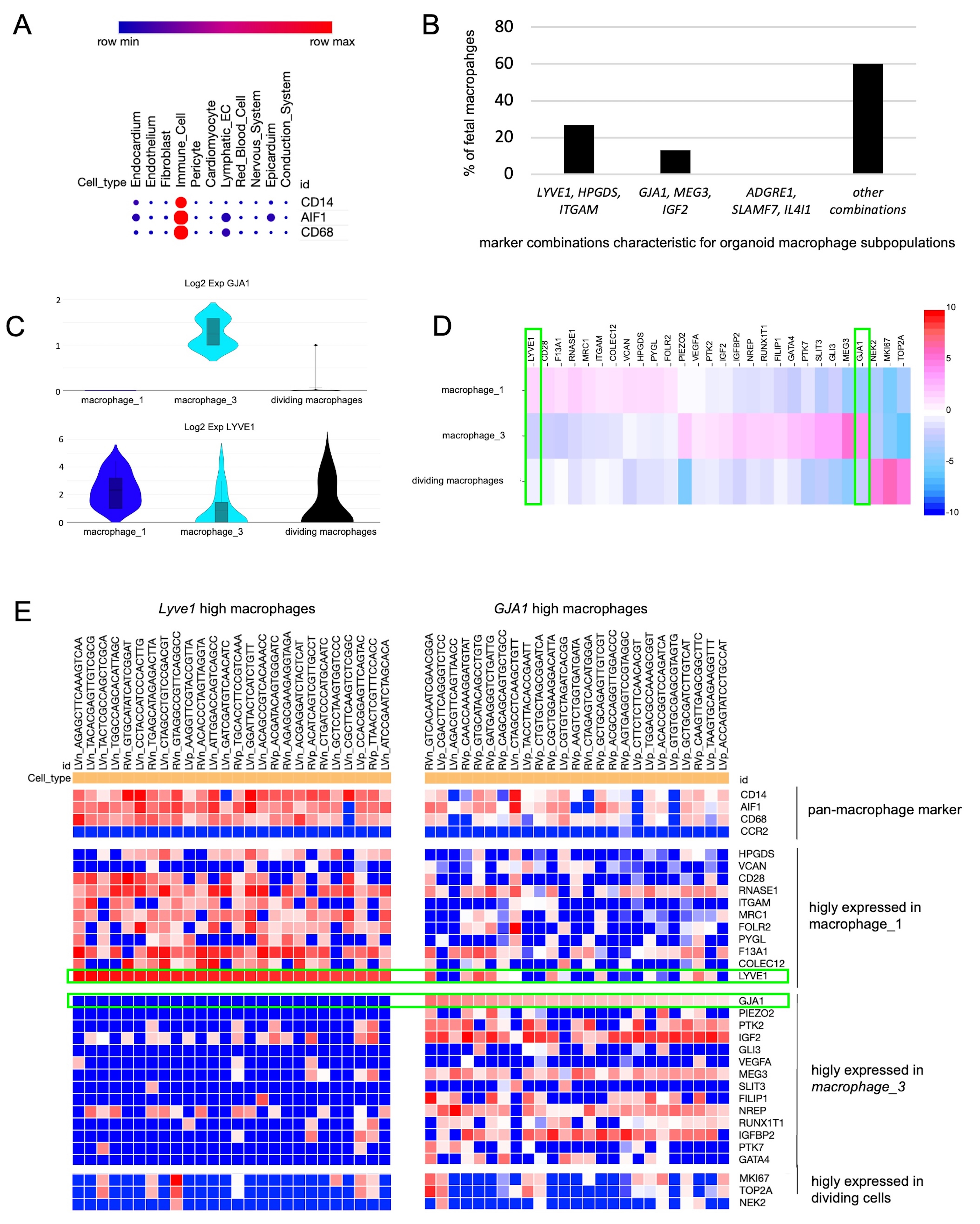

Supplement: Supplementary file 1 [file Image3.jpeg]

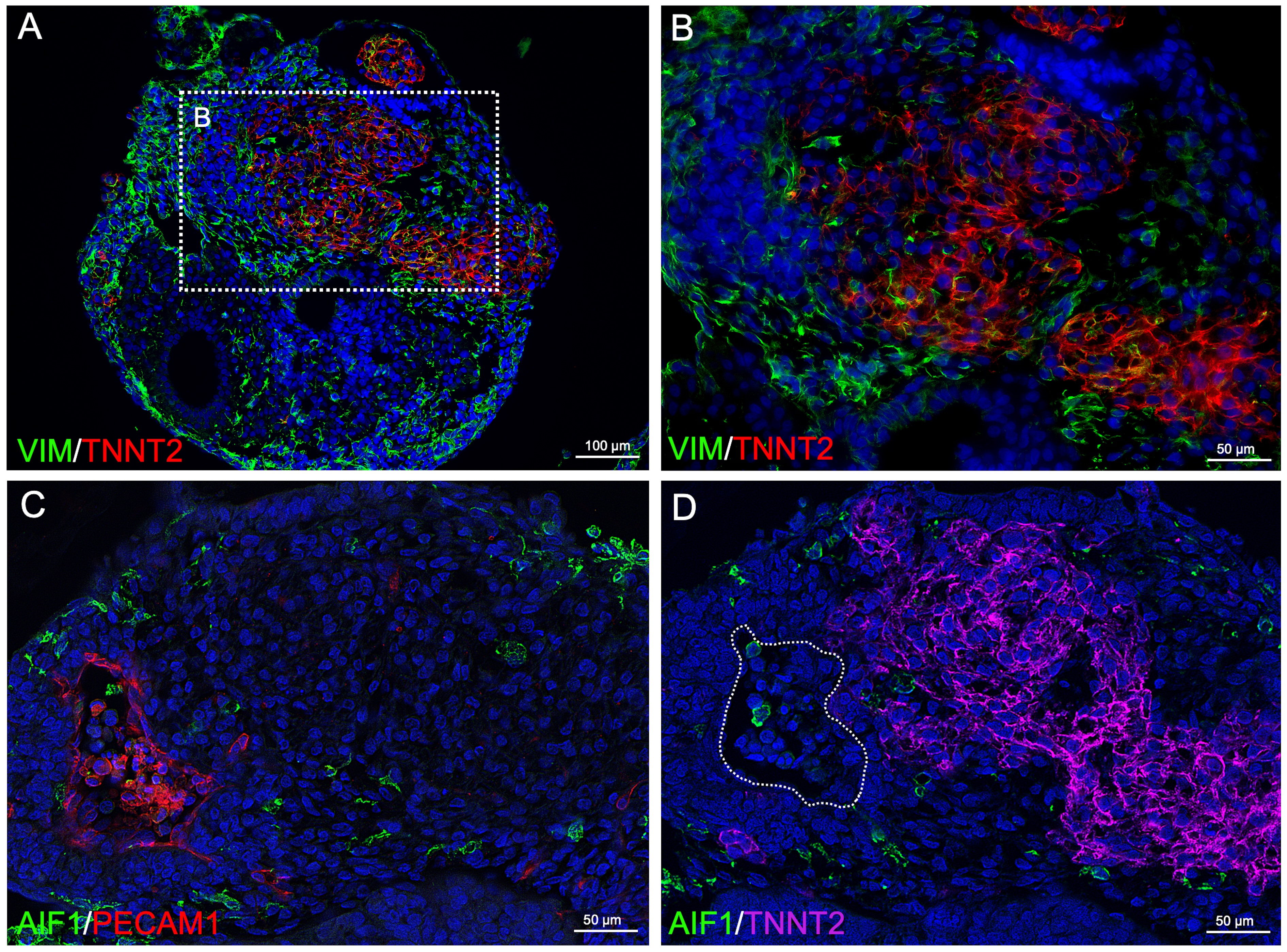

Supplement: Supplementary file 2 [file Image2.jpg]

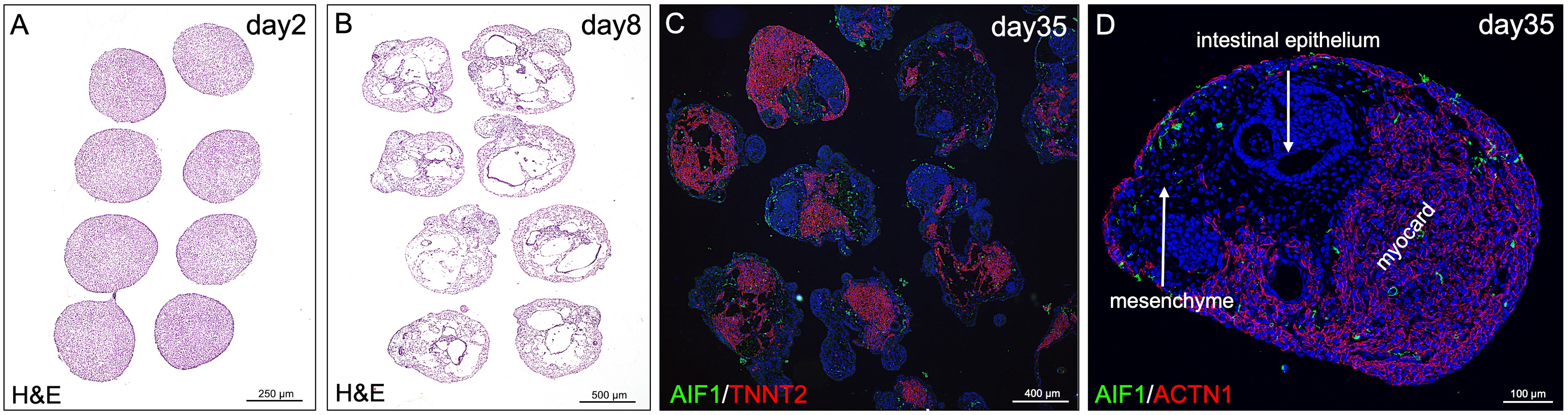

Supplement: Supplementary file 8 [file Image1.jpg]
